# Increased hypothalamic microglial activation after viral-induced pneumococcal lung infection is associated with excess serum amyloid A production

**DOI:** 10.1186/s12974-018-1234-1

**Published:** 2018-07-06

**Authors:** Hao Wang, Melissa Blackall, Luba Sominsky, Sarah J. Spencer, Ross Vlahos, Melissa Churchill, Steven Bozinovski

**Affiliations:** School of Health and Biomedical Sciences, RMIT University, PO Box 71, Bundoora, VIC 3083 Australia

**Keywords:** Microglia, Neuroinflammation, Pneumonia, Serum amyloid A, Resolvin-D1

## Abstract

**Background:**

It is well established that lung pathology and inflammation are more severe during respiratory infections complicated by the presence of both bacteria and viruses. Whilst co-infection can result in invasive pneumococcal disease and systemic inflammation, the neuroinflammatory consequences of co-infection are poorly characterised.

**Methods:**

In this study, we utilised a mouse co-infection model involving *Streptococcus pneumoniae* (*S. pneumoniae*) and influenza A virus (IAV) lung infection, and we also isolated microglia for ex vivo stimulation with pneumococcus or serum amyloid A (SAA).

**Results:**

Co-infection but not *S. pneumoniae* or IAV alone significantly increased the number of amoeboid-shaped microglia and expression of pro-inflammatory cytokines including tumour necrosis factor α (TNFα), interleukin-1β (IL-1β), interleukin-6 (IL-6), and C-C motif chemokine ligand-2 (CCL-2) in the hypothalamus. Pneumococcus was only detected in the hypothalamus of co-infected mice. In addition, the systemic inflammatory cytokines TNFα, IL-1β and IL-6 were not elevated in co-infected mice relative to IAV-infected mice, whereas SAA levels were markedly increased in co-infected mice (*p* < 0.05). SAA and its functional receptor termed formyl peptide receptor 2 (Fpr2) transcript expression were also increased in the hypothalamus. In mouse primary microglia, recombinant SAA but not *S. pneumoniae* stimulated TNFα, IL-1β, IL-6 and CCL-2 expression, and this response was completely blocked by the pro-resolving Fpr2 agonist aspirin-triggered resolvin D1 (AT-RvD1).

**Conclusions:**

In summary, lung co-infection increased the number of ‘activated’ amoeboid-shaped microglia and inflammatory cytokine expression in the hypothalamus. Whilst persistent pneumococcal brain infection was observed, SAA proved to be a much more potent stimulus of microglia than pneumococci, and this response was potently suppressed by the anti-inflammatory AT-RvD1. Targeting Fpr2 with pro-resolving eicosanoids such as AT-RvD1 may restore microglial homeostasis during severe respiratory infections.

**Electronic supplementary material:**

The online version of this article (10.1186/s12974-018-1234-1) contains supplementary material, which is available to authorized users.

## Background

Pandemic influenza A viruses (IAV) cause significant lung pathology consequent to an excessive host immune response that is associated with poor clinical outcome [1]. Clinically, many deaths attributable to pandemic IAV outbreaks have been associated with secondary bacterial complications driven by *Streptococcus pneumoniae* (*S. pneumoniae*) or the ‘pneumococcus’ which exacerbates the lung immunopathology [2]. Acute IAV lung infections that predispose to secondary pneumococcal super-infections can also result in invasion of the bacterium into other organs including the brain [3]. In addition to the pandemic super-infection setting, viral and bacterial co-infections occur frequently in susceptible people with underlying chronic lung disease such as COPD or asthma [4]. The chronology of co-infection in susceptible COPD and severe asthma patients is different from super-infections, as the lower airways can be chronically infected with pathogenic bacteria before they encounter a viral pathogen. Acute flares or COPD ‘exacerbations’ associated with bacterial and viral co-infections are clinically more severe as they result in greater lung function impairment and longer hospitalisations [4]. However, the central and neuroinflammatory consequences of bacterial and secondary acute viral co-infections have not been characterised.

In the classic pandemic super-infection setting, *S. pneumoniae* can enter the brain and proliferate in the central nervous system (CNS), and the ensuing inflammatory response can lead to brain injury and bacterial meningitis in severe cases [5]. Whilst meningitis rarely occurs in susceptible individuals, severe respiratory infections do routinely cause prolonged sickness behaviour including fever, malaise, fatigue, poor sleep quality and social withdrawal. The onset of sickness behaviour is mediated by the production of soluble inflammatory cytokines such interleukin (IL)-1β, IL-6 and tumour necrosis factor alpha (TNFα) produced at the primary site of lung infection that enter the circulation [6]. The production of peripheral cytokines can then act centrally to stimulate neuroinflammation through multiple pathways including activation of primary afferent nerves in the lung that project to the brain, activation of macrophage-like cells residing in the circumventricular organs and the choroid plexus brain regions characterised by highly permeable vasculature and overflow of systemic inflammation into the brain via cytokine transporters within the blood–brain barrier (BBB) (5). Peripheral administration of inflammatory cytokines induces symptoms of sickness including fever and activation of hypothalamic-pituitary-adrenal (HPA) axis. The paraventricular nucleus (PVN) of the hypothalamus is particularly active during infection, as this region indirectly stimulates adrenal glucocorticoid production. Whilst glucocorticoids can suppress peripheral cytokines production by blocking NFκB-mediated transcription, they can also promote central microglial activation and neuroinflammation [7].

Pro-inflammatory cytokines that enter the brain stimulate neuroinflammation by activating resident microglia [8, 9]. Systemic lipopolysaccharide (LPS) administration has been shown to markedly activate microglia, which then release pro-inflammatory cytokines, free radicals and proteases that can contribute to progressive neurodegeneration in the chronic setting [8, 9]. Peripheral production of inflammatory cytokines in response to viral and bacterial infection will also stimulate hepatic production of the acute phase protein serum amyloid a (SAA) [10]. Circulating levels of SAA markedly rise during infection and decline with clinical recovery [10]. We have previously shown that infectious exacerbations of chronic obstructive pulmonary disease (COPD) that are viral and bacterial in aetiology result in persistently elevated levels of circulating SAA [11]. We have also previously shown that circulating levels of serum SAA are increased in co-infected mice relative to mice infected with either *S. pneumoniae* or IAV alone [12]. SAA is a functional agonist for the Formyl peptide receptor 2 (Fpr2), which is expressed by multiple types of immune cells including neutrophils, monocytes, macrophages and microglia. This interaction promotes a pro-inflammatory state by simulating chemotaxis and expression of inflammatory cytokines [13, 14].

Fpr2 can also interact with an alternative class of lipid agonist known as resolvin-D1 (RvD1) and its stable aspirin-triggered epimer (AT-RvD1). AT-RvD1 allosterically modulates Fpr2 by actively opposing the actions of SAA to resolve lung inflammation and immunopathology [12, 13]. Whilst SAA is known to efficiently enter the brain from the circulation [15], its role in neuroinflammation in the context of respiratory co-infection is unexplored. In addition, as acute inflammation increases Fpr2 expression in primary microglia [16], pro-resolving mediators that target this receptor may represent an important target for modulating the function of microglia. To assess this, we have utilised our existing mouse co-infection model that exhibits excessive immunopathology in the lung [12]. In this study, we report that the pneumococcal 19F serotype was cleared from the lungs by 7 days; however, in co-infected mice, *S. pneumoniae* persisted in the lung and was also found in the brain. We also report that co-infection caused persistent microglial activation in the hypothalamus and identify SAA as a potent inflammatory stimulus of microglia that can be blocked by AT-RvD1.

## Methods

### Animals

Male C57BL/6J mice (8–10 weeks old) from the Animal Resources Centre (Perth, Australia) were housed at 22 ± 1 °C under normal 12 h light/dark cycle and fed a standard chow and water ad libitum. All experiments were approved by the Animal Ethics Committee of RMIT University (AEC #1509) and performed in compliance with the National Health and Medical Research Council (NHMRC) of Australia guidelines. Mice were divided into four groups: SAL (saline), SP (*S. pneumoniae*), IAV and SPIAV (co-infected). On day 0 of the experiment, mice from the SP and SPIAV groups received *S. pneumoniae* (serotype 19F, strain EF3030, 10^5^ CFU in 35 μL saline) intranasally under light isoflurane anaesthesia, whereas mice from the SAL and IAV groups received equivalent volume of saline. On day 1, mice from IAV and SPIAV groups received influenza A viruses (strain A/HKx31 (H3N2), 10^4^ PFU in 30 μL saline) intranasally, whereas mice from the SAL and SP groups received equivalent volume of saline. On day 7, mice were culled by intraperitoneal overdose of sodium pentobarbitone, and blood was collected from the vena cava. Prior to tissue collection, mice were perfused free of blood via right ventricle with 10 mL ice-cold PBS. Tissues including nasopharynx, olfactory epithelium, olfactory bulbs, hypothalamus and lungs were excised and snap-frozen for further analysis.

### Quantification of *S. pneumoniae* and influenza A virus

Quantitative real-time PCR (qPCR) was used to measure *S. pneumoniae* and IAV in target tissue. Briefly, bacterial DNA and viral RNA were isolated by homogenising tissue in Trizol (Life Technologies) using a TissueLyser (Qiagen) in accordance to the manufacturer’s instructions. *S. pneumoniae* DNA qPCR was performed using a commercial kit from Qiagen as per manufacturer’s instructions, and bacterial load was determined by using standard curve generated from a known quantity of pneumococci. RT-qPCR on polymerase A subunit (PA) gene of IAV was performed using TaqMan® Fast Virus 1-Step Master Mix as previously described [17]. Viral load was determined by using standard curve generated from a known quantity of IAV.

### ELISA assays

Serum was separated from whole blood using Microvette® 500 Z-Gel tubes (Sarstedt AG&CO). Serum IL-6, IL-1β, TNFα and SAA levels were determined using commercial ELISA kits (Life Technologies) according to the manufacturer’s instructions.

### Immunohistochemistry

Whole brains were dissected and fixed in 10% neutral buffered formalin. Brains were then processed, paraffin-embedded and sectioned coronally at a thickness of 5 μm. Sections were deparaffinised and antigens were retrieved by incubating sections in sodium citrate buffer (10 mM sodium citrate, 0.05% Tween 20, pH 6.0) at 95 °C for 20 min. After blocking in 5% bovine serum albumin (BSA) supplemented with 20% horse serum (Life Technologies), sections were incubated in ionized calcium-binding adapter molecule 1 (Iba-1) antibody (rabbit anti-mouse Iba-1, Wako Pure Chemical Industries, 1: 200), Glial fibrillary acidic protein (GFAP) antibody (rabbit anti-mouse GFAP, DAKO, Agilent Technologies, 1: 200) or TNFα antibody (goat anti-mouse, R&D Systems, 1:50) for 2 h at room temperature. This was followed by 30 min incubation in secondary antibody (AlexaFluor 488 goat anti-rabbit IgG or Alexa 594 donkey anti-goat IgG, Life Technologies, 1:200) and 2 min incubation in DAPI (Life Technologies, 1:2000 from 5 mg/mL stock). Alternatively, sections were incubated with SAA antibody (goat anti-mouse SAA, R&D Systems, 1:200) for 2 h at room temperature, followed by 1-h incubation in secondary antibody (donkey anti-goat IgG biotinylated antibody, R&D Systems, 1:200) and 1-h incubation in avidin-biotin horseradish peroxidase (HRP) complex (VECTASTAIN® Elite® ABC-HRP Kit, Vector Laboratories Ltd). After development in diaminobenzidine (DAB) solution, sections were counter stained with haematoxylin.

Images were taken from hypothalamic paraventricular nucleus (PVN) containing sections (between − 0.70 and − 0.94 mm from the bregma) or hippocampus containing sections (3–4 sections per mouse for Iba-1 and GFAP staining; 1 section per mouse for SAA staining) using a VS120 Olympus slide scanner (Olympus) or a BX53 System Microscope (Olympus). Images were then analysed using Cellsens software (Olympus). Iba-1/GFAP immunoreactivity was determined by measuring the number of pixels above a set threshold value and expressed as a percentage of total pixels within the chosen region of interest. Density of microglia and astrocytes is expressed as an average of area faction of Iba-1and GFAP respectively. Number of activated microglia in PVN (amoeboid-shaped), identified morphologically with enlarged soma and fewer processes (< 4), was manually counted by two independent assessors who were blinded to group treatments. The values from each of the assessors were averaged to derive data presented in this study.

### Microglia isolation and treatments

Naïve male C57BL/6J mice (8–10 weeks old) were perfused free of blood via right ventricle with 10 mL ice-cold PBS, and whole brains were collected. Cells were isolated from these brains using a Neural Tissue Dissociation Kit (Miltenyi Biotec) according to manufacturer instructions. Myelin was removed by centrifuging isolated cells in 37% Percoll solution (GE Healthcare Life Sciences). The cell pellets were re-suspended in culture medium (DMEM/F-12 supplemented with 10% fetal bovine serum and 100 U/mL Penicillin-Streptomycin, Life Technologies) and dispersed into 24-well plates. After 30 min incubation in 5% CO_2_ at 37 °C, cells in suspension were removed and enriched (> 90%) microglia were obtained by further washing off the non-adhering cells with culture medium as previously described [18]. Microglia were then pre-treated with aspirin-triggered resolvin D1 (AT-RvD1, 10 nM, Cayman Chemical) or medium (Veh) for 40 min, followed by *S. pneumoniae* (MOI of 1), recombinant human Apo-SAA1 (SAA, 1 μM, PeproTech) or PBS (Veh) for 4 h. Medium was then removed. Cells were washed with PBS and collected for further analysis.

### Reverse transcriptase quantitative PCR (RT-qPCR) for gene expression analysis

Total RNA was extracted and purified from tissue or primary microglia using RNeasy kit (Qiagen), from which cDNA was prepared using High Capacity cDNA Kit (Life Technologies) as previously described [12]. qPCR was performed using bioinformatically validated Taqman primers/probes, namely TNFα, IL-1β, IL-6, CCL-2, Fpr2 and SAA1 (Life Technologies). The threshold cycle values (Ct) were normalized to a reference gene (glyceraldehyde phosphate dehydrogenase or GAPDH for isolated microglia and phosphoglycerate kinase 1 or PGK1 for brain tissue samples) and the relative fold change determined by the ΔΔCt value as previously described [12].

### Data analysis

Data are presented as the mean ± SEM. All data were statistically analysed using GraphPad Prism 7.0 (Graphpad, San Diego, CA). Where detailed and appropriate, two-tailed Student’s unpaired *t* tests or one-way analyses of variance (ANOVA) with Bonferroni’s post hoc tests were used. *p* < 0.05 was considered to be statistically significant.

## Results

### Hypothalamic neuroinflammation was only present in co-infected mice

**Fig. 1:**
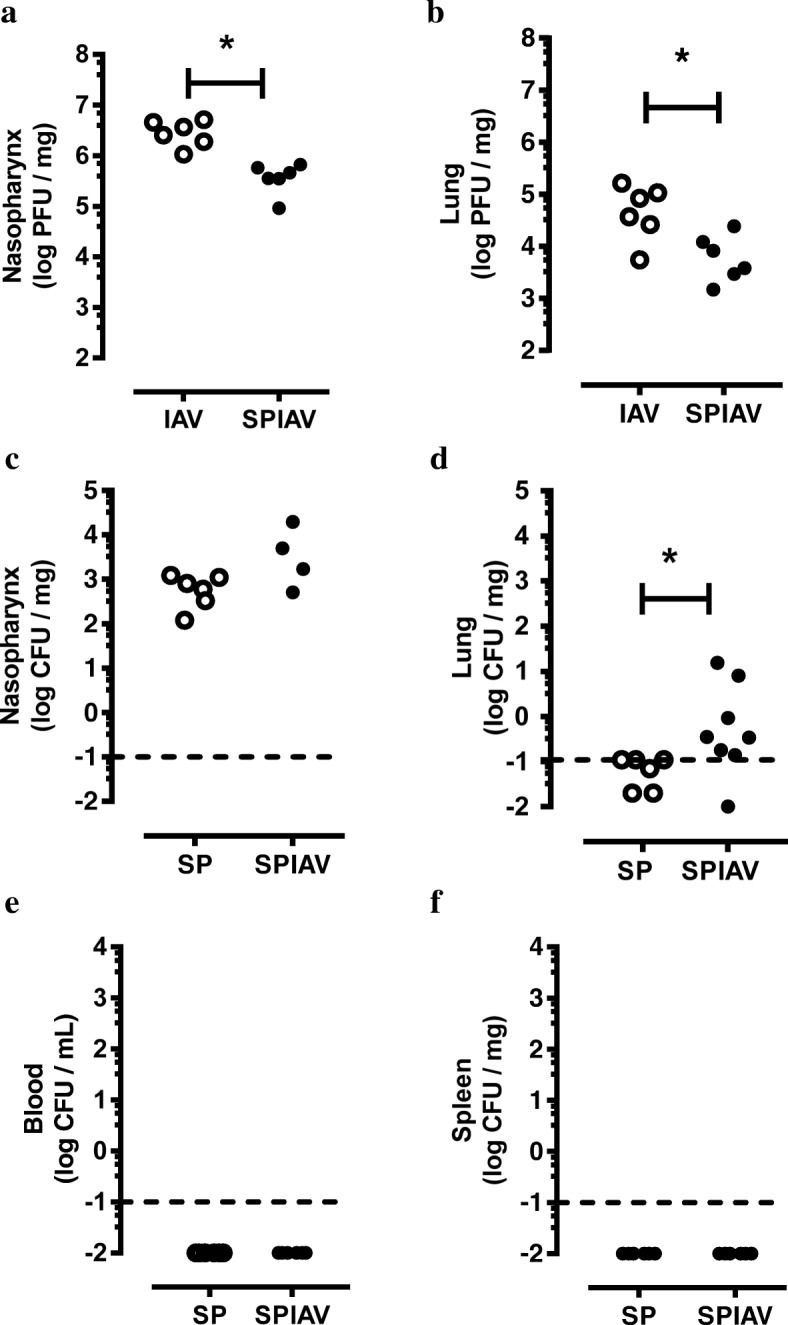
Co-infection in the lung causes pneumococcal brain infection. Mice were infected intranasally with *S. pneumoniae* (SP, serotype 19F/strain EF3030, 10^5^ CFU) and/or influenza A virus (IAV, strain HKx31, H3N2, 10^4^ PFU) on day 0 and day 1 respectively. On day 7, mice were culled, yielding four groups consisting of control (SAL), single pathogen infection (SP and IAV), and co-infection (SPIAV). Viral titres were assessed in nasopharynx (**a**) and lung (**b**), whilst bacterial titres were measured in in nasopharynx (**c**), lung (**d**), blood (**e**) and spleen (**f**). Dashed lines denote detection limits. *n* = 4–6; *p < 0.05, two-tailed Student’s unpaired *t* tests

We utilised our previously published co-infection model [12], where mice are infected with pneumococcus first and then subsequently infected with IAV to model the chronology of infection in people with underlying chronic lung conditions such as COPD. We firstly evaluated IAV load in the upper and lower airways by RT-qPCR. At day 7 post-pneumococcal infection (day 6 post-IAV infection), we observed a high viral load in the nasopharynx tissue in IAV-infected mice, which was approximately 1-log lower in the *S. pneumoniae*-IAV (SPIAV) co-infected mice (Fig. 1a, *p* < 0.05). The IAV load was approximately 2-log lower in the lung tissue relative to the nasal tissue, and again lung viral levels were significantly lower by 1-log in co-infected mice relative to IAV-infected mice (Fig. 1b).

Using the same tissue samples, pneumococcal levels were also determined by qPCR, where high pneumococcal carriage was detected in the nasopharynx of *S. pneumoniae* and co-infected mice, with no significant difference between these two groups (Fig. 1c). This strain of *S. pneumoniae* (EF3030) was effectively cleared from the lungs in the absence of IAV infection, with all the samples being at or below the limit of detection (Fig. 1d). In co-infected mice, pneumococcal lung load was significantly increased relative to *S. pneumoniae* infection alone, where 6/8 lung samples were above the limit of detection for this sensitive assay (Fig. 1d). These data are consistent with our previously published findings [12] that quantified pneumococcal load by viable count in the BAL fluid (BALF). In addition, blood and spleen samples were screened by RT-qPCR, and no detection of pneumococcus was observed (Fig. 1e, f).

**Fig. 2:**
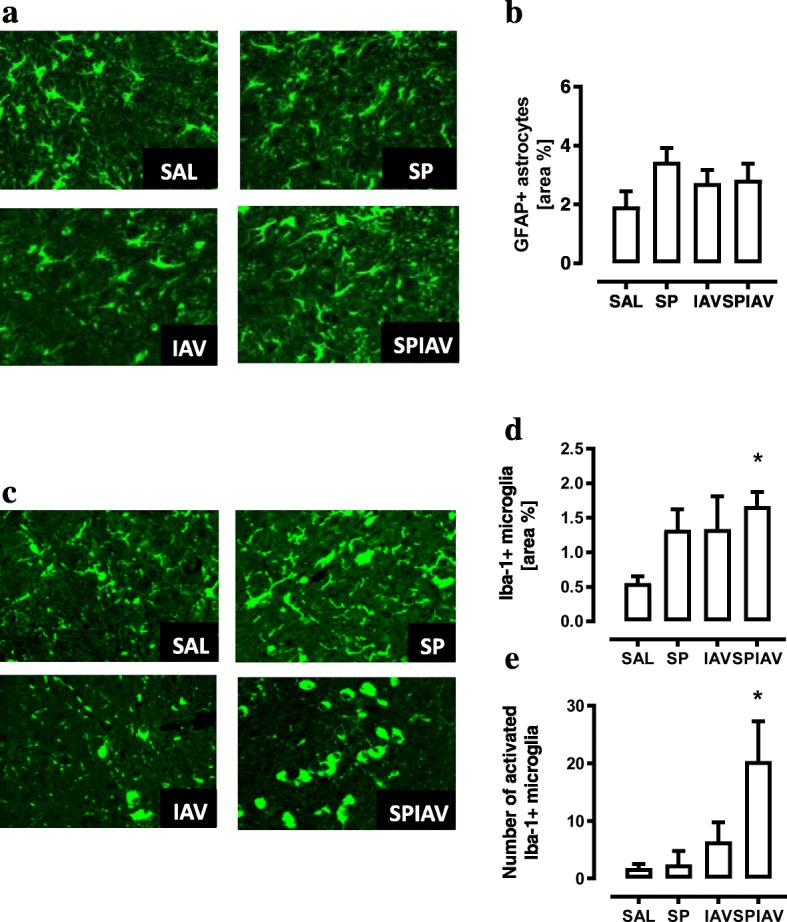
Co-infection in the lung activates microglia. Brain sections containing paraventricular ventricle (PVN) from saline (SAL), *S. pneumoniae* (SP), influenza A virus (IAV) or co-infection (SPIAV)-treated mice were immunolabelled for astrocytic marker GFAP (**a**) and GFAP-positive area were quantified (**b**). Microglia were stained with Iba-1 antibody (**c**), and Iba-1-positive area was quantified (**d**). Number of ameboid microglia within the PVN was manually counted (**e**). *n* = 5–6 with 3–4 sections from each brain; *p < 0.05, one-way ANOVA compared to SAL

Since the HPA axis is highly active during infection and systemic inflammation, immunofluorescent staining for astrocytes and microglia was performed within the PVN region of the hypothalamus. The percentage area staining for GFAP-positive cells was not statistically different between all the control and infection groups (Fig. 2a, b). In contrast, staining for Iba-1-positive cells revealed an emergence of ‘activated’ amoeboid-shaped microglia in the PVN region of co-infected brains relative to the saline-treated group (Fig. 2c). The area positive for Iba-1 immunoreactivity was quantified, and only co-infected mice were significantly increased relative to saline-treated mice (Fig. 2d). More strikingly, quantitative assessment of the number of amoeboid microglia within the entire sampled PVN revealed a significant 10-fold increase in numbers of amoeboid microglial cells compared to saline control (Fig. 2e). We also evaluated astrocytes and microglia in the hippocampus (Additional file 1: Figure S1 and S2). No difference in percentage area positive for GFAP staining (astrocytes) or Iba-1 staining (microglia) was detected in the hippocampus across the treatment groups, and we did not observe the presence of amoeboid microglia in this region.

**Fig. 3:**
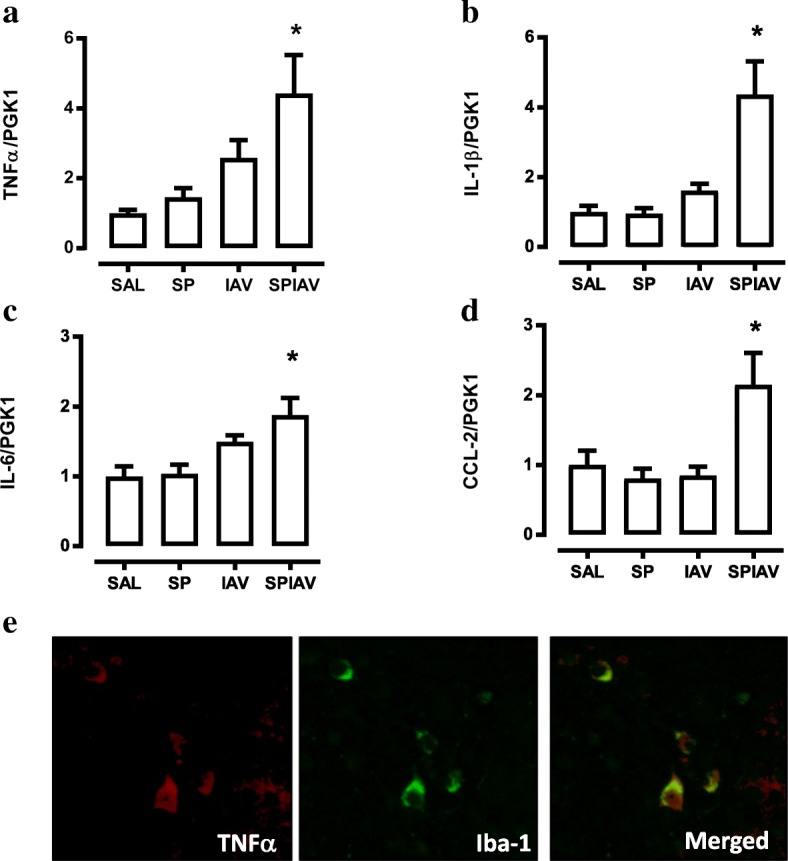
Co-infection in the lung upregulates inflammatory cytokines/chemokines in the hypothalamus. The hypothalamus region of the brains was dissected from mice with saline (SAL), *S. pneumoniae* (SP), influenza A virus (IAV) or co-infection (SPIAV) treatment. Gene expression of **a** TNFα, **b** IL-1β, **c** IL-6 and **d** CCL-2 was measured. *n* = 6–8; *p < 0.05, one-way ANOVA compared to SAL. **e** We next performed immunohistochemical staining on hypothalamic sections to identify sources of TNFα. TNFα-positive cells were stained red; Iba-1-positive cells were stained green; and double-positive cells in the merged image appeared yellow

To determine whether the presence of activated microglia in the hypothalamus was associated with an increase in inflammatory cytokine expression, we performed RT-qPCR on hypothalamus. TNFα and IL-1β transcript levels were only significantly increased in the hypothalamus of co-infected mice relative to saline-treated mice (Fig. 3a, b, four-fold increase, *p* < 0.05). In addition, IL-6 and CCL-2 levels were increased in co-infected mice by two-fold relative to SAL (Fig. 3b, c). No significant change in gene expression was observed in mice infected with IAV or pneumococcus alone (Fig. 3a–d). In addition, we assessed inflammatory gene expression in the hippocampus (Additional file 1: Figure S3), and whilst TNFα was significantly increased in co-infected mice, IL-6, IL-1β and CCL-2 were not altered by single- or co-infection treatment. We also performed co-staining experiments to identify potential sources of TNFα, which demonstrated Iba-1 positive amoeboid microglia co-localised with TNFα in hypothalamus of co-infected mice (Fig. 3e). In contrast, we did not detect any co-localisation between TNFα and GFAP positive cells (astrocytes) within the hypothalamus (Additional file 1: Figure S4).

### Pneumococcal brain infection is elevated in co-infected mice

**Fig. 4:**
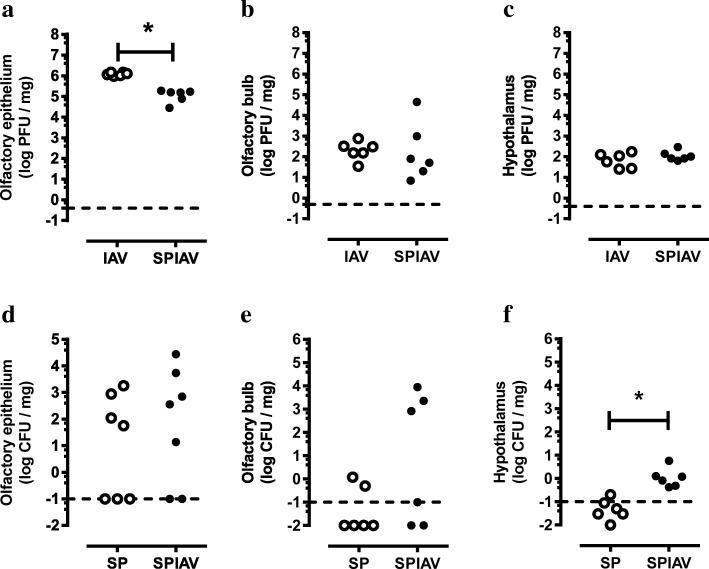
Co-infection facilitates greater pneumococcal entry into the CNS. Olfactory epithelium, olfactory bulb and hypothalamus were dissected from mice with saline (SAL), *S. pneumoniae* (SP), influenza A virus (IAV) or co-infection (SPIAV) treatment. Titres of influenza A viruses and *S. pneumoniae* in these tissues were measured in (**a**–**c**) and (**d**–**f**) respectively. Dashed lines denote detection limits. *n* = 6; *p < 0.05, two-tailed Students unpaired *t* tests

To determine whether the hypothalamic neuroinflammation is associated with pathogen entry into the brain, we next examined viral and bacterial load in the olfactory region and the hypothalamus. Since the olfactory epithelium is located in the roof of the posterior nasal cavity, we observed a 1-log reduction in viral loads in isolated olfactory epithelium (Fig. 4a) as seen in the nasopharynx tissue (Fig. 1a). IAV was also detected within the isolated olfactory bulb, with levels 4-log lower than in the olfactory epithelium in IAV and co-infected mice (Fig. 4b). Low levels of IAV were detected in the hypothalamus of IAV-infected mice, and this did not differ from co-infected mice (Fig. 4c). Levels of pneumococcal DNA in the olfactory epithelium were similar to those detected in the nasopharyngeal tissue, with no significant difference between *S. pneumoniae* and co-infected mice (Fig. 4d). Levels of pneumococcal DNA were inconsistently detected in the olfactory bulb with 2/6 and 3/6 mice harbouring pneumococcus above the limits of detection in *S. pneumoniae* and co-infected mice respectively (Fig. 4e). However, co-infection did result in significantly higher pneumococcal load in the hypothalamus relative to *S. pneumoniae* alone, with 6/6 detectable in co-infection and only 1/6 in *S. pneumoniae* alone (Fig. 4f).

### Hypothalamic SAA and Fpr2 transcript expression is elevated in co-infected mice

**Fig. 5:**
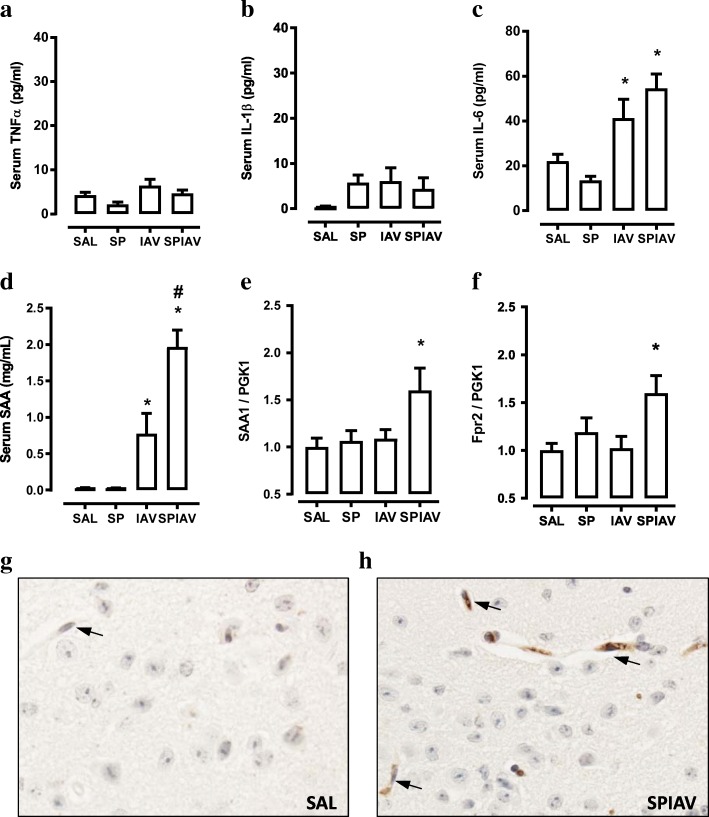
Co-infection increased SAA and Fpr2 expression in the hypothalamus. Serum levels of **a** TNFα, **b** IL-1β, **c** IL-6 and **d** SAA from mice treated with saline (SAL), *S. pneumoniae* (SP), influenza A virus (IAV) or co-infection (SPIAV). Gene expression of **e** SAA1 and **f** Fpr2 in the hypothalamus. **g–h** Representative immunohistochemical images of SAA staining in the PVN region of the brain. Arrows denote the vasculature. *n* = 4–6; *p < 0.05, one-way ANOVA compared to SAL; #*p* < 0.05, one-way ANOVA compared to IAV

To determine whether hypothalamic neuroinflammation is associated with systemic inflammation, TNFα, IL-1β and IL-6 levels were measured in serum by quantitative ELISA (Fig. 5a–c). At day 7 post-pneumococcal infection, circulating TNFα and IL-1β were not significantly different across the groups. IL-6 levels were significantly increased in IAV-infected mice; however, this was not further increased in co-infected sera. We have previously shown that circulating levels of SAA are markedly upregulated in the co-infection setting at day 7 [12]. We have replicated these findings in this cohort and show that serum SAA levels in co-infected mice (1.97 ± 0.23 mg/mL) were significantly increased (*p* < 0.05, ANOVA) compared to saline-treated (0.03 ± 0.01 mg/mL), *S. pneumoniae*-infected (0.03 ± 0.00 mg/mL) or IAV-infected (0.78 ± 0.28 mg/mL) mice (Fig. 5d).

In addition to circulating SAA levels, SAA transcript in the hypothalamus showed a 50% increase in co-infected mice only (Fig. 5e). Furthermore, transcript levels of Fpr2, a receptor for SAA that regulates inflammatory and resolution signalling circuits, were significantly increased in co-infected mice (Fig. 5f). Immunohistochemistry staining for SAA in the PVN demonstrated little immunoreactivity in brain sections from saline-treated mice (Fig. 5g). Staining in co-infected brain sections identified intense vascular immunoreactivity that is consistent with elevated circulating SAA levels (Fig. 5h, arrows).

### SAA induced inflammation in primary microglial cells that were blocked by AT-RvD1

**Fig. 6:**
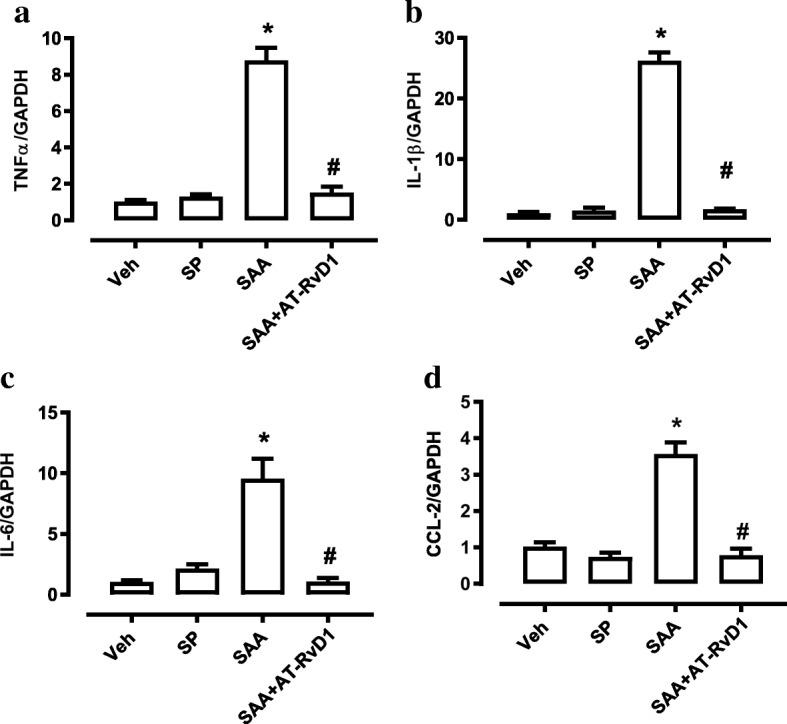
SAA increased cytokine expression in primary microglia, which was blocked by AT-RvD1. Gene expression of **a** TNFα, **b** IL-1β, **c** IL-6 and **d** CCL-2 from primary microglia cultured in DMEM/F-12 medium and stimulated with *S. pneumoniae* (MOI of 1), recombinant SAA (1 μM), recombinant SAA with the addition of aspirin-triggered resolving D1 (RvD1, 10 nM) or vehicle (Veh) for 4 h. *n* = 2–6; *p < 0.05, one-way ANOVA compared to Veh; #*p* < 0.05, one-way ANOVA compared to SAA

To evaluate if *S. pneumoniae* or SAA can directly activate microglia and is thus capable of contributing to the neuroinflammatory response to co-infection, primary microglia were isolated and challenged with *S. pneumoniae* or recombinant SAA. Interestingly, after 4 h of incubation, this serotype of *S. pneumoniae* did not stimulate proinflammatory gene expression at MOI of 1, as shown in Fig. 6a–d. In contrast, recombinant SAA markedly induced (a) TNFα, (b) IL-1β, (c) IL-6 and (d) CCL-2 gene expression by 4 to 26-fold. Of significance, the pro-inflammatory action of SAA was completely blocked by treatment with AT-RvD1 (Fig. 6a–d).

## Discussion

In this study, we demonstrate that hypothalamic neuroinflammation was significantly increased as a consequence of pneumococcal and influenza A viral co-infection of the lower airways. The neuroinflammatory response was associated with an increase in the number of activated amoeboid microglia and inflammatory gene expression including IL-1β, IL-6, TNFα and CCL-2. The hypothalamus appeared to be particularly responsive to lung co-infection, as we did not observe any difference in microglial or astrocyte morphology in the hippocampus and whilst TNFα expression was increased, IL-6, IL-1β and CCL-2 expression were unaltered. This suggests that brain regions in close proximity to circumventricular organs that lack a contiguous BBB, such as the PVN, were exposed to a circulating factor that was increased in co-infected mice. Alternatively, pneumococcal entry into the hypothalamus may be driving this inflammatory response. We also show that pneumococcal brain entry occurs more frequently in the co-infection setting, and this appears to occur directly via the nasal cavity. Whilst hematogenous routes are known to facilitate pathogen entry into the CNS, there is evidence that the nasal cavity can provide an alternative route [19]. The mouse-adapted pathogenic strains of IAV have been shown to enter the brain via olfactory nerves, and this stimulates a rapid cytokine and anti-viral response within the olfactory bulb [20]. Our findings show that although low levels of IAV were detected in the hypothalamus, the immunological response to IAV was not present at day 7, likely due to the resolution process.

A secondary consequence of IAV infection was the persistent detection of *S. pneumoniae* in the hypothalamus of co-infected mice. *S. pneumoniae* can be readily detected in the CNS after bacteraemia [21]; however, the less invasive 19F serotype used in this study was not detected in the bloodstream or other organs. It is therefore plausible that the pneumococci in the hypothalamus were directly transported from the nasal cavity and IAV may have facilitated entry into the CNS via its neuraminidase activity [22, 23]. Since the detection of *S. pneumoniae* was quite low in the hypothalamus (1.6 CFU/mg tissue) and this serotype did not stimulate microglial activation under in vitro conditions at MOI of 1 CFU per cell, we investigated whether a specific component of the systemic inflammatory response was capable of contributing to neuroinflammation.

Notably, circulating SAA was significantly elevated in co-infected mice, in contrast to other inflammatory cytokines such as IL-1β, IL-6 and TNFα in the serum. Elevated serum SAA levels were also associated with intense SAA immunoreactivity in co-infected brain sections, which was localised to the vasculature within the hypothalamus. In the chronic setting, excessive SAA production is a precursor to secondary amyloidosis that can cause serious complications [24], and amyloid deposits can also accumulate within the brains of patients with systemic amyloidosis, particularly around circumventricular organs that lack a contiguous BBB [25]. The acute SAA isoforms have been shown to readily cross the intact BBB, where radiolabelled SAA traversed the endothelial barrier to enter the brain parenchyma [15]. In a recent study, a liver-specific SAA over-expressing transgenic mouse model was used to evaluate whether acute circulating SAA can contribute to neurodegenerative disorders [26]. Excessive production of SAA in this transgenic model resulted in accumulation of SAA in the brain, and this stimulated an increase in microglial cell numbers and expression of pro-inflammatory cytokines including IL-6 and TNFα [26]. In addition, chronic elevation of circulating SAA resulted in behavioural abnormalities that are indicative of depressive-like behaviour and social withdrawal [26]. Importantly, our study provides a clinically relevant infectious model of excessive peripheral SAA production that leads to microglial pro-inflammatory activity and neuroinflammation.

The increase in circulating SAA and hypothalamic SAA transcript in our study was accompanied by increased expression of Fpr2 in the hypothalamus. Since a previous study demonstrated that SAA potently stimulates microglia relative to astrocytes [27], and as we did not see changes in astrocyte density in co-infected mice, we focused on defining how SAA mechanistically coordinates microglial activation. We specifically utilised the pro-resolving AT-RvD1, as Fpr2 is the only known receptor for this eicosanoid in mice and it is proven to be effective in various inflammatory disease models [13, 28, 29]. Using isolated microglia, we demonstrate that SAA potently stimulated expression of inflammatory cytokine genes. We also demonstrate for the first time that SAA-dependent microglial activation can be effectively blocked by AT-RvD1.

## Conclusions

In summary, we show for the first time that high level of circulating SAA induced by viral and pneumococcal lung infection can lead to microglial activation and upregulation of proinflammatory cytokines in the hypothalamus in mouse brain. Our data suggests the pro-resolving actions of specialised lipid mediators that target Fpr2 such as AT-RvD1 should be considered as an alternative strategy to reduce this excessive and persistent microglial activation and neuroinflammation during severe respiratory co-infections.

## Additional file

Additional file 1: **Figure S1.** Co-infection in the lung does not activate astrocytes in the hippocampus. Brain sections containing hippocampus from saline (SAL), *S. pneumoniae* (SP), influenza A virus (IAV) or co-infection (SPIAV)-treated mice were immunolabelled for astrocytic marker GFAP (upper panel) and GFAP-positive area were quantified (lower panel). *n* = 5–6 with 3–4 sections from each brain. **Figure S2.** Co-infection in the lung does not activate microglia in the hippocampus. Brain sections containing hippocampus from saline (SAL), *S. pneumoniae* (SP), influenza A virus (IAV) or co-infection (SPIAV)-treated mice were immunolabelled for astrocytic marker GFAP (upper panel), and GFAP-positive area were quantified (lower panel). *n* = 5–6 with 3–4 sections from each brain. **Figure S3.** Co-infection selectively increases TNFα gene expression in the hippocampus. The hippocampal region of the brains were dissected from mice treated with saline (SAL), *S. pneumoniae* (SP), influenza A virus (IAV) or co-infection (SPIAV). Gene expression of (A) TNFα, (B) IL-1β, (C) IL-6 and (D) CCL-2 was measured. *n* = 6–8; *p < 0.05, one-way ANOVA compared to SAL. **Figure S4.** TNFα does not co-localise with GFAP in the hypothalamus of co-infected mice. Brain sections containing hypothalamus from co-infected mice (SPIAV) were doublestained for TNFα (red) and astrocytic marker GFAP (green) and images were merged for colocalisation. (PDF 539 kb)

## Abbreviations

AT-RvD1: Aspirin triggered-resolvin D1
BBB: Blood-brain barrier
CCL-2: C-C Motif Chemokine Ligand 2
CNS: Central nervous system
Fpr2: Formyl peptide receptor 2
IAV: Influenza A virus
IL-1β: Interleukin-1 β
IL-6: Interleukin-6
PVN: Paraventricular nucleus
SAA: Serum amyloid A
SP: Streptococcus pneumoniae
TNFα: Tumour necrosis factor α
