# Supplementary material for: Increased hypothalamic microglial activation after viral-induced pneumococcal lung infection is associated with excess serum amyloid A production
Source: J Neuroinflammation. 2018 Jul 6;15:200. doi: 10.1186/s12974-018-1234-1 (PMC6035471; doi:10.1186/s12974-018-1234-1)
Supplement: Supplementary file 1 — Figure S1. Co-infection in the lung does not activate astrocytes in the hippocampus. Brain sections containing hippocampus from saline (SAL), S. pneumoniae (SP), influenza A virus (IAV) or co-infection (SPIAV)-treated mice were immunolabelled for astrocytic marker GFAP (upper panel) and GFAP-positive area were quantified (lower panel). n = 5–6 with 3–4 sections from each brain. Figure S2. Co-infection in the lung does not activate microglia in the hippocampus. Brain sections containing hippocampus from saline (SAL), S. pneumoniae (SP), influenza A virus (IAV) or co-infection (SPIAV)-treated mice were immunolabelled for astrocytic marker GFAP (upper panel), and GFAP-positive area were quantified (lower panel). n = 5–6 with 3–4 sections from each brain. Figure S3. Co-infection selectively increases TNFα gene expression in the hippocampus. The hippocampal region of the brains were dissected from mice treated with saline (SAL), S. pneumoniae (SP), influenza A virus (IAV) or co-infection (SPIAV). Gene expression of (A) TNFα, (B) IL-1β, (C) IL-6 and (D) CCL-2 was measured. n = 6–8; *p < 0.05, one-way ANOVA compared to SAL. Figure S4. TNFα does not co-localise with GFAP in the hypothalamus of co-infected mice. Brain sections containing hypothalamus from co-infected mice (SPIAV) were doublestained for TNFα (red) and astrocytic marker GFAP (green) and images were merged for colocalisation. (PDF 539 kb) [file 12974_2018_1234_MOESM1_ESM.pdf]

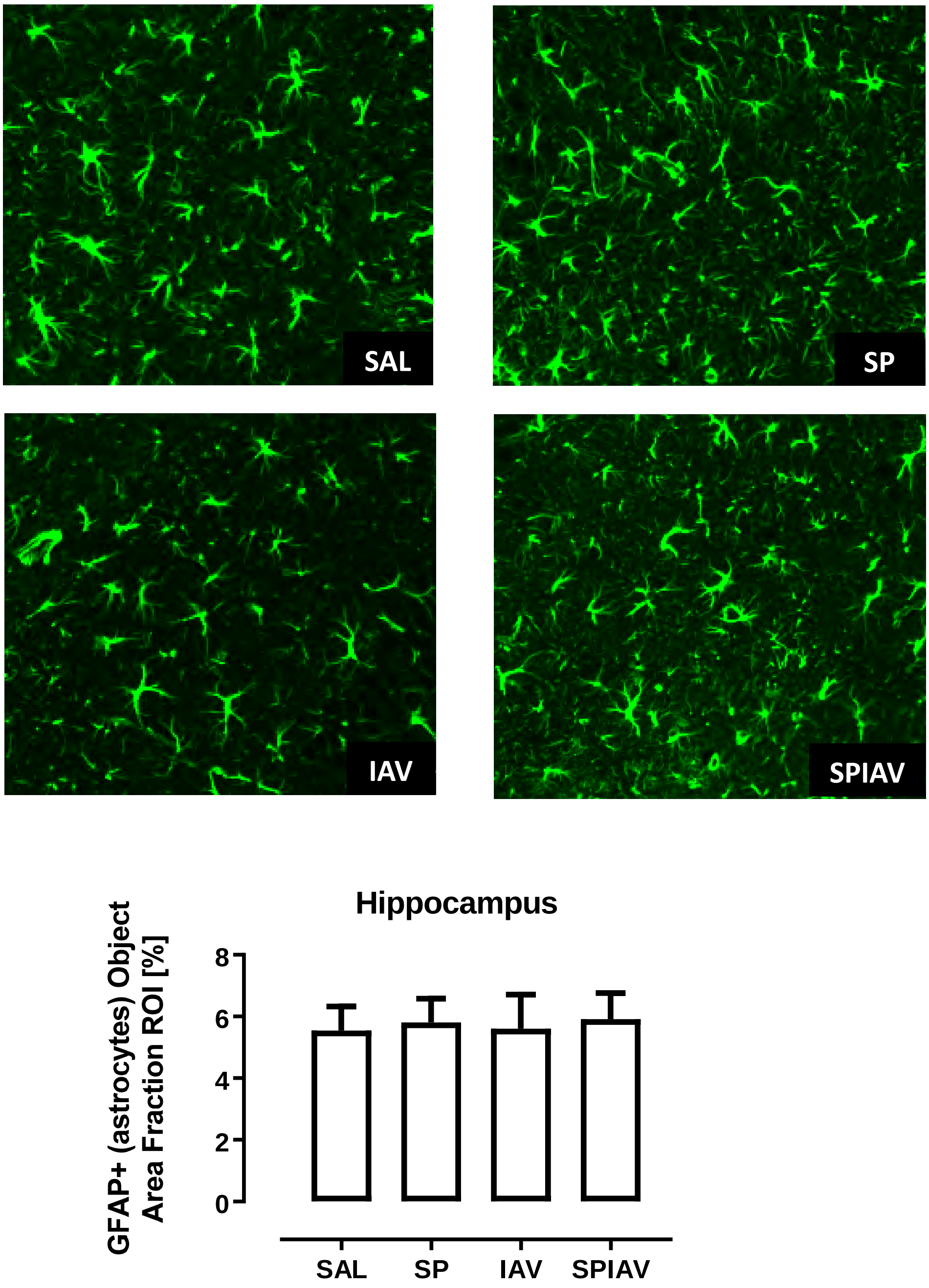

Figure S1. Co-infection in the lung does not activate astrocytes in the hippocampus. Brain sections containing hippocampus from saline (SAL), *S. pneumoniae* (SP), influenza A virus (IAV) or co-infection (SPIAV)-treated mice were immunolabelled for astrocytic marker GFAP (upper panel) and GFAP-positive area were quantified (lower panel). n = 5 – 6 with 3 – 4 sections from each brain.

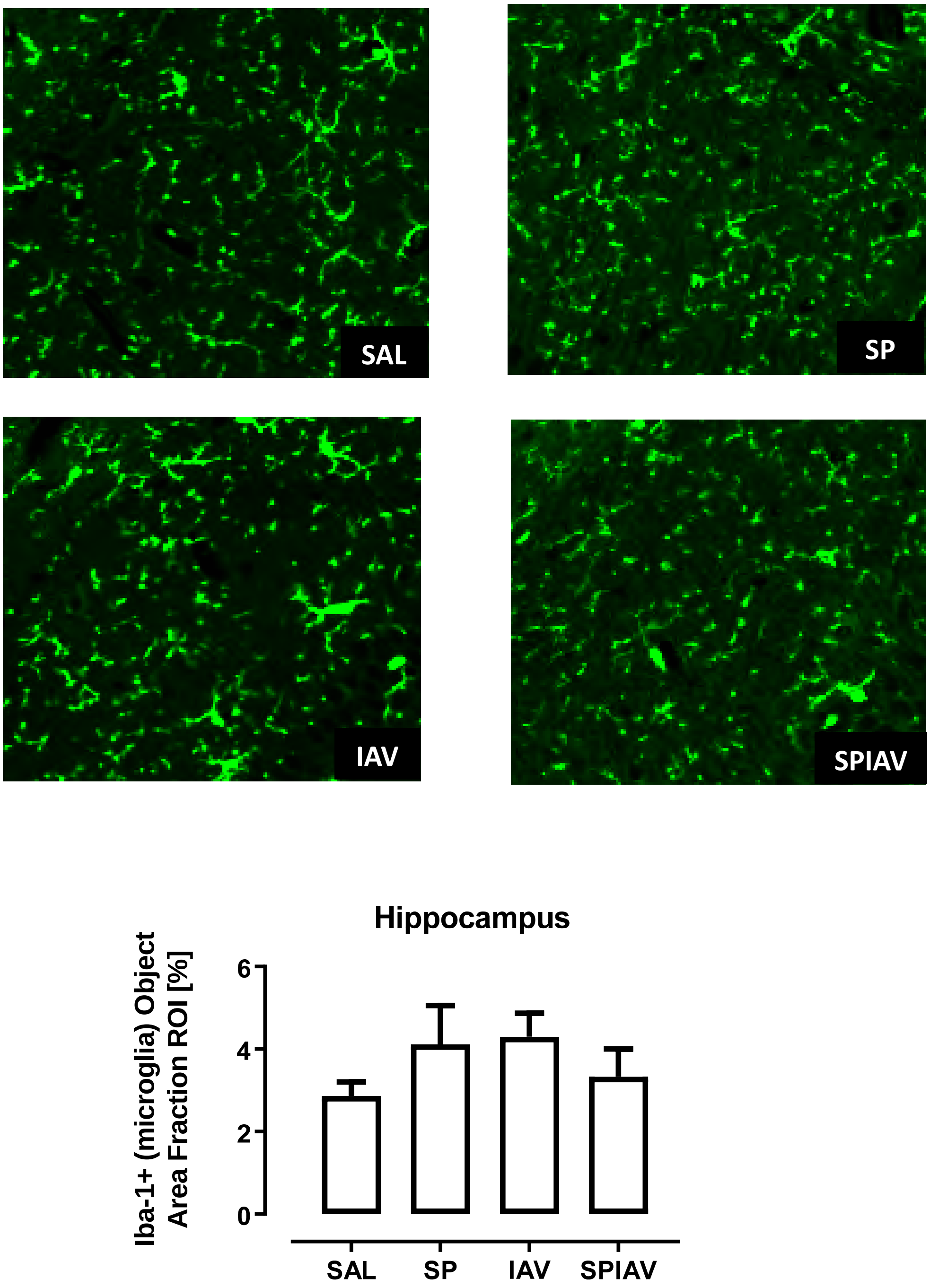

Figure S2. Co-infection in the lung does not activate microglia in the hippocampus. Brain sections containing hippocampus from saline (SAL), *S. pneumoniae* (SP), influenza A virus (IAV) or co-infection (SPIAV)-treated mice were immunolabelled for astrocytic marker GFAP (upper panel) and GFAP-positive area were quantified (lower panel). n = 5 – 6 with 3 – 4 sections from each brain.

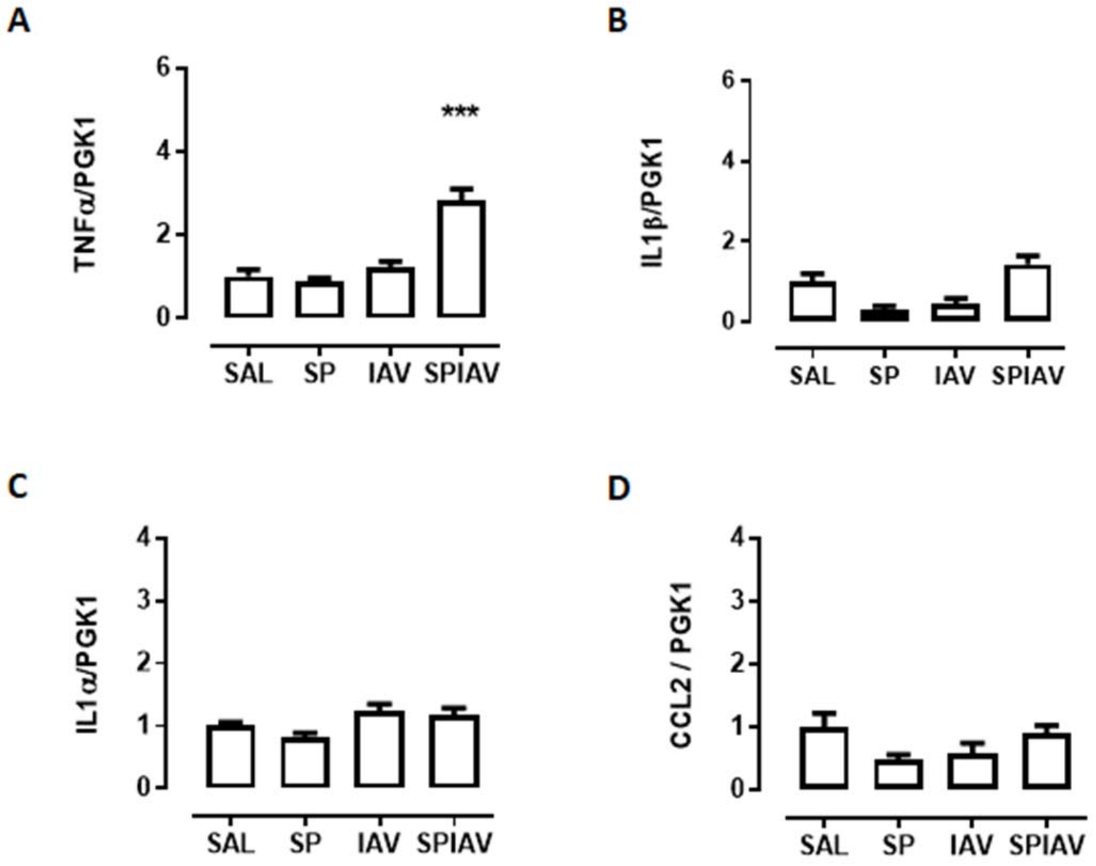

Figure S3. Co-infection selectively increases TNFα gene expression in the hippocampus. The hippocampal region of the brains were dissected from mice treated with saline (SAL), *S. pneumoniae* (SP), influenza A virus (IAV) or co-infection (SPIAV). Gene expression of (**A**) TNFα, (**B**) IL-1β, (**C**) IL-6 and (**D**) CCL-2 was measured. n = 6 – 8; *p<0.05, one-way ANOVA compared to SAL.

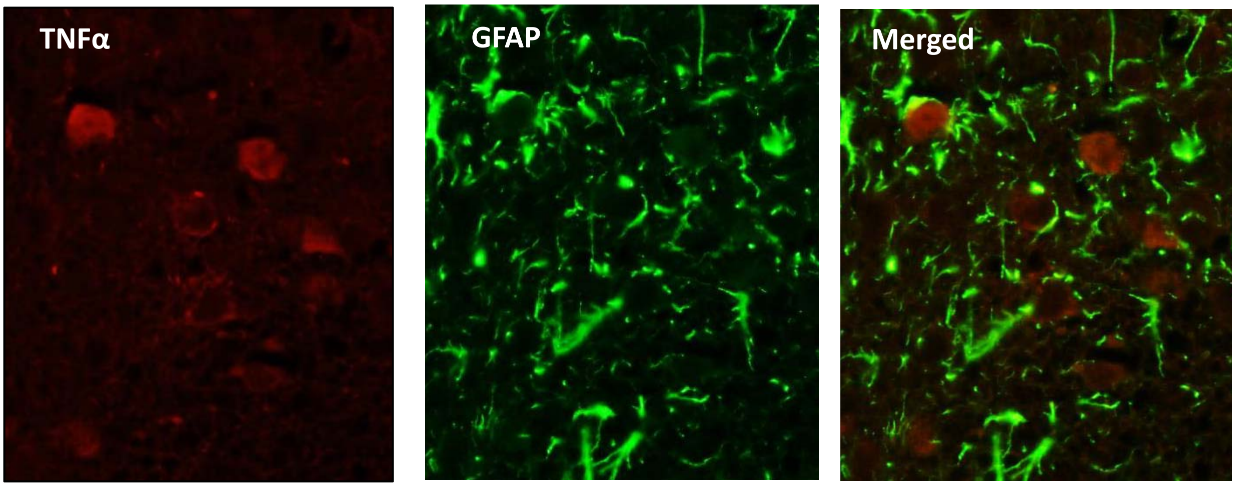

Figure S4. TNFα does not co-localise with GFAP in the hypothalamus of co-infected mice. Brain sections containing hypothalamus from co-infected mice (SPIAV) were double-stained for TNFα (red) and astrocytic marker GFAP (green) and images were merged for co-localisation.
